# Effect of time interval from diagnosis to treatment for cervical cancer on survival: A nationwide cohort study

**DOI:** 10.1371/journal.pone.0221946

**Published:** 2019-09-04

**Authors:** Chao-Ping Chen, Pei-Tseng Kung, Yueh-Hsin Wang, Wen-Chen Tsai

**Affiliations:** 1 Department of Health Services Administration, China Medical University, Taichung, Taiwan, R.O.C; 2 Department of Orthopaedics, Taichung Veterans General Hospital, Taichung, Taiwan, R.O.C; 3 Jen-Teh Junior College of Medicine, Nursing and Management, Miaoli, Taiwan, R.O.C; 4 Department of Healthcare Administration, Asia University, Taichung, Taiwan, R.O.C; 5 Department of Medical Research, China Medical University Hospital, China Medical University, Taichung, Taiwan, R.O.C; Universidade Estadual de Maringa, BRAZIL

## Abstract

**Objectives:**

Despite the ease of health care access and the waiver of copayments for cancer patients, treatment is delayed in a small proportion of Taiwanese patients diagnosed with cervical cancer. In this study, we explored the relationship between the time interval from diagnosis to treatment and survival in cervical cancer patients.

**Material and methods:**

The study was a retrospective population-based observational study conducted between 2004 and 2010. In Taiwan, 12,020 patients were newly diagnosed with cervical cancer from 2004 to 2010, and 9,693 patients (80.6%) were enrolled in our final analysis.

**Results:**

Most of the patients received treatment within 90 days of diagnosis (n = 9,341, 96.37%). After adjustment for other variables, patients who received treatment between 90 and 180 days and >180 days after diagnosis had a 1.33 (95% CI: 1.02–1.72, *P* < 0.05) and 1.36 (95% CI: 1.12–1.65, *P* < 0.05) times higher risk of death, respectively, than those who received treatment within 90 days. Kaplan–Meier analysis showed that the patients treated after 90 days from diagnosis had a lower overall survival rate than those treated within 90 days. In analysis stratifying the patients according to their initial tumor stage, namely stages I and II and stage III and IV, the time interval from diagnosis to treatment remained a significant prognosticator in those who received treatment >180 days after diagnosis.

**Conclusion:**

A longer interval between diagnosis and treatment is associated with poorer prognosis among cervical cancer patients.

## Introduction

Cervical cancer is the fourth most common cancer among women, and 528,000 new cases of cervical cancer were diagnosed worldwide in 2012. [1] In Taiwan, in 2015, approximately 4014 women were diagnosed with cervical cancer, and more than 661 women died of the disease, making cervical cancer the seventh most common cause of cancer-related death among Taiwanese women and a significant health concern in Taiwan. [2] In 1995, the Department of Health of Taiwan started providing reimbursement to women over the age of 30 years who underwent annual pap smear examinations. Consequently, the standardized cervical cancer mortality rate decreased by 68% between 1995 and 2015; with a decrease in the mortality rate from 11 to 3.5 per 100,000 people, respectively. The cervical cancer standardized incidence rate also decreased by 62% between 1995 and 2013, with a decrease in the incidence rate from 25 to 9.5 per 100,000 people, respectively. [2]

Prompt diagnosis and treatment are advocated for controlling the progression of cancer. The stage of cervical cancer is the most crucial factor in the choice of treatment. The Taiwan Cooperative Oncology Group (TCOG) and National Health Research Institutes have published “Therapy Guideline of Cervical Cancer” (revised in 2000) and “Clinical Practice Guideline of Gynaecologic Oncology” (revised in 2011) for cervical cancer screening and treatment. Although a cohort study reported the effectiveness of the screening program in reducing the mortality of cervical cancer, the positive effects of the screening program were not observed in elderly women because of treatment delays. [3] Several studies have also reported poor prognosis when medication or treatment is delayed. [4–6] However, no systematic review has reached a conclusive agreement regarding the effect of treatment delay in different types of cancer. [7, 8] Commonly, delayed treatment is categorized into four components as patient delay, healthcare provider delay, referral delay, and system delay. [9] Patients with cervical cancer have experienced delayed diagnosis and treatment regardless of country or institution. [10] A second opinion seeking or time-consuming pathological results waiting may also lead to delay in treatment. [11, 12] Poor access to healthcare services is the main reason of delayed treatment in low- and middle-income countries. [13] Patient delays have a more crucial role in developing countries. [14]

The National Health Insurance (NHI) program covers 99.9% of the population of Taiwan, and under this insurance program, copayments for cancer patients is waived. [15] The NHI administration has also included 93% of Taiwan’s health services organizations as NHI-contracted health care providers as of the end of 2014. [15] The Taiwan Cancer Registry (TCR), a population-based cancer registry, was founded in 1979. The registry is organized and funded by the Ministry of Health and Welfare. The Taiwan Cancer Registry Database (TCRD) records data of all types of cancer diagnosed and treatments in patients in Taiwan. The accuracy in the diagnosis of major diseases listed in the NHIRD, such as acute coronary syndrome and ischemic stroke, has been validated in previous studies. [16,17] Despite the ease of access to health care services and the waiver of copayments for cancer patients, treatment is delayed in a small proportion of Taiwanese patients diagnosed with cervical cancer. From the public health perspective, understanding patient behavior is critical for encouraging patients to seek appropriate intervention. Determining the distribution of durations until initial treatment is useful to health officials who formulate strategies for encouraging patients to receive timely treatment. In this study, the factors related to treatment delay in cervical cancer patients were identified, and the effects of treatment delay on their survival were examined.

## Materials and methods

The study was a retrospective population-based observational study. We extracted the claims data of patients newly diagnosed with cervical cancer between 2004 and 2010 from the TCRD. This study was approved by the Institutional Review Board of Cheng Ching Hospital Chung Kang Branch (IRB number: HP150003) and was conducted in accordance with the Helsinki Declaration. Identification information of all patients was omitted prior to analysis.

### Selection of participants

We identified all patients who had been diagnosed with cervical cancer from 2004 to 2010 (International Classification of Diseases, Oncology, Third Edition [ICD-O-3] site codes: C53.0–C53.9) as the patient group. Cervical cancer cases are distinguished from other primary disease sites by corresponding ICD-O-3 site codes. We included patients with histology of SCC only (ICD-O-3 histology codes 8010–8671, 8940–8941). The follow-up end point was set as December 31, 2012. The accuracy of diagnosis was validated based on ICD-O-3 codes and inclusion in the TCRD. The patients were excluded if they died within 1 month of their confirmed diagnosis, had carcinoma in situ, had distant metastases at diagnosis and only received palliative treatment or hospice care, had an unknown stage of cervical cancer, had multiple primary cancers, and had incomplete data in the NHIRD and TCRD.

### Description of variables

Demographic data, including age at the confirmation of diagnosis, were documented. The time interval between diagnosis and treatment was defined as the time from the date of diagnosis (date of biopsy, which confirmed malignancy) until the date of initiation of the first treatment in the patients. All cancer patients are listed in the NHI catastrophic illness or injury registry file, which contains 30 categories of patients with any severe illness or injury, including cancer, chronic renal failure, systemic lupus erythematosus, and type I diabetes, as defined by the NHI. [18] The National Cancer Registry contains information on the cervical cancer clinical staging system (stages I–IV) developed by the International Federation of Gynecology and Obstetrics; this information is representative and is related to all histological types of cervical cancer. [19] The urbanization level ranged from highly developed urban cities (level 1) to remote districts (level 7). [20] The number of services provided by primary hospitals was divided into three categories, namely low, medium, and high, based on the quartile (low: lowest quartile, medium: second and third quartile, and high: highest quartile). Hospital ownership was divided into public and private sectors. The degree of comorbidity was categorized into three levels according to the Charlson comorbidity index (CCI), as modified by Deyo. [21] Other variables included patients’ monthly income and hospital level (medical centers, regional hospitals, and others).

### Main outcome measurement

The primary outcome measured was overall survival. Follow-up duration was defined as the duration from the date of diagnosis to the date of death or follow-up endpoint, which was the end of 2012. Death was identified as the withdrawal of a patient from the NHI program and was validated by linking the administrative data set with the Taiwan Death Registry.

### Statistical analysis

We calculated descriptive statistics for general data presentation. Comparisons of nominal or ordinal variables between patients who were alive or dead were conducted using the chi-square test, whereas continuous variables were analyzed using the Student’s *t* test. In addition, the survival time was calculated using the Kaplan–Meier method and was stratified by various tumor stages to investigate the effects of the time interval between diagnosis and treatment on overall survival in cervical cancer patients. Furthermore, a univariate Cox proportional hazard regression was used to analyze the prognostic factors for survival. Finally, modified Cox proportional hazard models were used to analyze the hazard ratios of the death of patients with various treatment delay durations after adjustment for age and other variables. All statistical analyses were performed using SAS software, version 9.2 (SAS Institute Inc., Cary, NC, USA). A *P* value of <0.05 was considered statistically significant, and all tests were two-sided.

## Results

In Taiwan, 12,020 patients were newly diagnosed with cervical cancer from 2004 to 2010. In this study, 1,774 (14.8%) patients had missing information on cancer stage and 290 (2.4%) patients who had incomplete records. In addition, multiple primary cancers or distant metastasis receiving palliative treatment or hospice care after cervical cancer diagnosed were observed in 260 (2.2%) patients. After excluding the abovementioned patients and repeated patients, 9,693 (80.6%) patients were enrolled in our final analysis. The average age at the time of diagnosis was 56.7 (±14.3) years, and the average follow-up period was 53.8 (±30.5) months. Most of the patients had undergone treatment within 90 days of diagnosis (n = 9,341, 96.4%), 152 patients (1.6%) received treatment between 91 and 180 days after diagnosis, and 200 patients (2.1%) received treatment >180 days after diagnosis (including patients without confirmed time interval and treatment initiation). Most of the patients presented with stages I and II (n = 7,480, 77.2%); 2213 (22.8%) patients presented with stages III and IV. Other detailed descriptive data are provided in Table 1.

**Table 1:** Descriptive statistics of cervical cancer patients with different time interval from diagnosis to treatment.

		Interval from cancer diagnosis to treatment			
Variables	Total	≤ 90 days	91~180 days	> 180 days	P value
	N (%)	N (%)	N (%)	N (%)	
**Total number**	9,693 (100%)	9,341 (96.37%)	152 (1.57%)	200 (2.06%)	-
**Age** (years)					<0.001
≤ 44	1,980 (20.43%)	1,908 (96.36%)	32 (1.62%)	40 (2.02%)	
45~54	2,920 (30.12%)	2,845 (97.43%)	44 (1.51%)	31 (1.06%)	
55~64	1,906 (19.66%)	1,855 (97.32%)	20 1.05%)	31 (1.63%)	
65~74	1,533 (15.82%)	1,473 (96.09%)	23 (1.50%)	37 (2.41%)	
≥ 75	1,354 (13.97%)	1,260 (93.06%)	33 (2.44%)	61 (4.51%)	
**Mean age** (y±sd)	56.68±14.28	56.53±14.14	58.19±17.07	62.40±17.39	<0.001
**Monthly salary** (NT dollars)					0.391
Low-income	123 (1.27%)	116 (94.31%)	4 (3.25%)	3 (2.44%)	
≤ 17280	572 (5.90%)	554 (96.85%)	8 (1.40%)	10 (1.75%)	
17281~22800	5,065 (52.25%)	4,868 (96.11%)	89 (1.76%)	108 (2.13%)	
22801~28800	1,826 (18.84%)	1,773 (97.10%)	19 (1.04%)	34 (1.86%)	
28801~36300	755 (7.79%)	721 (95.50%)	14 (1.85%)	20 (2.65%)	
36301~45800	660 (6.81%)	634 (96.06%)	10 (1.52%)	16 (2.42%)	
≥ 45801	692 (7.14%)	675 (97.54%)	8 (1.16%)	9 (1.30%)	
**Urbanization**					0.179
Level 1	2,736 (28.23%)	2,652 (96.93%)	34 (1.24%)	50 (1.83%)	
Level 2	3,001 (30.96%)	2,885 (96.13%)	51 (1.70%)	65 (2.17%)	
Level 3	1,603 (16.54%)	1,547 (96.51%)	29 (1.81%)	27 (1.68%)	
Level 4	1,353 (13.96%)	1,304 (96.38%)	18 (1.33%)	31 (2.29%)	
Level 5	219 (2.26%)	204 (93.15%)	6 (2.74%)	9 (4.11%)	
Level 6	378 (3.90%)	359 (94.97%)	10 (2.65%)	9 (2.38%)	
Level 7	403 (4.16%)	390 (96.77%)	4 (0.99%)	9 (2.23%)	
**CCI score**					<0.001
≤ 3	8,576 (88.48%)	8,284 (96.60%)	136 (1.59%)	156 (1.82%)	
4~6	774 (7.99%)	737 (95.22%)	12 (1.55%)	25 (3.23%)	
≥ 7	343 (3.54%)	320 (93.29%)	4 (1.17%)	19 (5.54%)	
**Other Catastrophic illness**					0.076
No	9,380 (96.77%)	9,044 (96.42%)	148 (1.58%)	188 (2.00%)	
Yes	313 (3.23%)	297 (94.89%)	4 (1.28%)	12 (3.83%)	
**Cancer stage**					<0.001
Stage I	5,092 (52.53%)	4,916 (96.54%)	92 (1.81%)	84 (1.65%)	
Stage II	2,388 (24.64%)	2,318 (97.07%)	29 (1.21%)	41 (1.72%)	
Stage III	1,323 (13.65%)	1,273 (96.22%)	18 (1.36%)	32 (2.42%)	
Stage IV	890 (9.18%)	834 (93.71%)	13 (1.46%)	43 (4.83%)	
**Hospital level**					<0.001
Medical centers	7,533 (77.72%)	7,303 (96.95%)	111 (1.47%)	119 (1.58%)	
Regional hospitals	2,064 (21.29%)	1,965 (95.20%)	40 (1.94%)	59 (2.86%)	
District hospitals	71 (0.73%)	58 (81.69%)	0 (0.00%)	13 (18.31%)	
Others	25 (0.26%)	15 (60.00%)	1 (4.00%)	9 (36.00%)	
**Hospital ownership**					0.235
Public	2,829 (29.19%)	2,737 (96.75%)	35 (1.24%)	57 (2.01%)	
Private	6,864 (70.81%)	6,604 (96.21%)	117 (1.70%)	143 (2.08%)	
**Hospital services volume**					<0.001
Low	2,436 (25.13%)	2,310 (94.83%)	42 (1.72%)	84 (3.45%)	
Middle	4,821 (49.74%)	4,660 (96.66%)	73 (1.51%)	88 (1.83%)	
High	2,436 (25.13%)	2,371 (97.33%)	37 (1.52%)	28 (1.15%)	

N = number, y = years, sd = standard deviation, NT dollars = New Taiwan dollars.

When the patients were stratified according to the time interval from diagnosis to treatment, variables such as patients’ survival status, age, CCI, and cancer stage; hospital level; and service volume of hospital were significant among the three groups. The patients aged ≥65 years who had advanced-stage cancer and high CCI scores tended to start treatment later. Furthermore, the average follow-up period was shorter in the patients who started treatment >180 days after diagnosis. Moreover, on average, the patients who received treatment in private hospitals had a shorter time interval from diagnosis to treatment than those treated in public hospitals. Finally, the patients who received treatment in hospitals with low or medium service volumes had a longer mean time interval from diagnosis to treatment than did those treated in hospitals with high service volumes. However, no significant differences were observed in patients’ monthly income, patients’ urbanization level, and hospital level among the three groups. Detailed data are presented in Table 1.

The results of analysis of the related factors and effect of treatment delay on survival in the cervical cancer patients are presented in Table 2. After adjustment for other variables, the risk of death in the patients who started their treatment after an interval of 91~180 days and >180 days was 1.33 (95% CI: 1.02–1.72, *P* < 0.05) and 1.36 (95% CI: 1.12–1.65, *P* < 0.05) times higher, respectively, than that in the patients who had started treatment within 90 days. The effects of other factors on survival were also analyzed. Compared with the reference age group, namely the patients aged ≤44 years, the patients aged 65–74 years and ≥75 years exhibited 1.15 (95% CI: 1.00–1.31, *P* < 0.05) and 2.14 (95% CI: 1.89–2.43, *P* < 0.05) times higher likelihood of death, respectively. The presence of other catastrophic illness or injury significantly affected the risk of death in cervical cancer patients (*P* < 0.05). Comorbidity in cervical cancer patients was positively correlated with mortality risk. Compared with the reference group with CCI scores of ≤3, the adjusted hazard ratio of death increased from 1.59 to 2.16 as CCI scores increased from 4–6 to ≥7 (*P* < 0.05). Mortality risk also increased with advanced cancer stage. Compared with cancer stage I, the adjusted hazard ratio of stages II, III, and IV increased significantly to 2.34, 4.15, and 8.84 (*P* < 0.05), respectively. The patients who received treatment in private hospitals exhibited a lower likelihood of death than did those who received treatment in public hospitals (*P* < 0.05). The risk of death was significantly lower in hospitals with middle and high service volume than in hospitals with low service volumes (*P* < 0.05). The urbanization level of the residential area and monthly income among patients and hospital level did not significantly affect mortality risk in cervical cancer patients (*P* > 0.05).

**Table 2:** Cox proportional hazard model for the related factors and effect of interval from cancer diagnosis to treatment on survival of patients with cervical cancer.

	Unadjusted HR	P value	Adjusted HR	95% CI	P value
**Interval from cancer diagnosis to treatment**					
≤ 90 days (ref.)					
91~180 days	1.26	0.078	1.33	1.02–1.72	0.035
> 180 days	2.22	<0.001	1.36	1.12–1.65	0.002
**Age** (years)					
≤ 44 (ref.)					
45~54	1.29	<0.001	1.01	0.90–1.14	0.854
55~64	1.38	<0.001	0.93	0.82–1.07	0.308
65~74	1.80	<0.001	1.15	1.00–1.31	0.043
≥ 75	3.96	<0.001	2.14	1.89–2.43	< .001
**Monthly salary** (NT dollars)					
Low-income (ref.)					
≤ 17280	0.79	0.128	1.06	0.74–1.51	0.745
17281~22800	0.98	0.907	1.08	0.78–1.48	0.653
22801~28800	0.98	0.910	1.08	0.78–1.50	0.629
28801~36300	0.83	0.271	1.04	0.74–1.47	0.833
36301~45800	0.80	0.216	0.95	0.67–1.35	0.762
≥ 45801					
**Urbanization**					
Level 1 (ref.)					
Level 2	1.04	0.392	1.01	0.92–1.11	0.867
Level 3	1.13	0.037	1.01	0.90–1.13	0.916
Level 4	1.11	0.075	1.01	0.89–1.14	0.890
Level 5	1.24	0.079	1.16	0.91–1.47	0.232
Level 6	1.54	<0.001	1.19	1.00–1.42	0.055
Level 7	1.23	0.023	1.06	0.88–1.27	0.532
**CCI score**					
≤ 3 (ref.)					
4~6	2.51	<0.001	1.59	1.43–1.77	< .001
≥ 7	4.93	<0.001	2.16	1.88–2.48	< .001
**Other Catastrophic Illness**					
No (ref.)					
Yes	2.02	<0.001	1.61	1.37–1.89	< .001
**Cancer stage**					
Stage I (ref.)					
Stage II	2.63	<0.001	2.34	2.12–2.58	< .001
Stage III	4.59	<0.001	4.15	3.73–4.61	< .001
Stage IV	11.49	<0.001	8.84	7.92–9.86	< .001
**Hospital level**					
Medical centers (ref.)					
Regional hospitals	1.35	<0.001	0.97	0.86–1.10	0.634
District hospitals	2.63	<0.001	1.31	0.94–1.82	0.116
Others	1.54	0.156	0.95	0.52–1.75	0.880
**Hospital ownership**					
Public (ref.)					
Private	0.94	0.100	0.82	0.75–0.90	< .001
**Hospital services volume**					
Low (ref.)					
Middle	0.70	<0.001	0.78	0.69–0.88	< .001
High	0.63	<0.001	0.69	0.60–0.79	< .001

N = number, y = years, sd = standard deviation, NT dollars = New Taiwan dollars.

We further analyzed the average survival time by characteristics (Table 3). For convenience, the patients were grouped according to their initial tumor stage: stages I & II and stages III & IV. At the end of 2012, 77.93% of patients in stage I & stage II survived whereas only 38.14% of patients in stage III and stage IV survived. The average follow-up time of survival patients was 67.93±24.79 and 60.21±24.59 months in stages I & II and stages III & IV, respectively. The survival patients exhibited significantly higher follow-up time than other patients (P < 0.05). Detailed data are presented in Table 3.

**Table 3:** Average follow-up time or survival time (months) for the cervical cancer patients.

Variables		Stage I&II					Stage III&IV				
		Alive		Death		P value	Alive		Death		P value
		N	follow-up time (m±sd)	N	follow-up time (m±sd)		N	follow-up time (m±sd)	N	follow-up time (m±sd)	
**Total number**		5,829	67.93±24.79	1,651	30.44±21	<0.001	844	60.21±24.59	1,369	18.01±15.81	<0.001
**Interval from cancer diagnosis to treatment**											
	≤ 90 days	5,668	67.62±24.73	1,566	30.47±21.01	<0.001	826	60.21±24.57	1,281	18.07±15.83	<0.001
	91~180 days	84	77.73±24.67	37	26.43±20.80	<0.001	10	59.70±24.37	21	19.45±15.52	<0.001
	>180 days	77	79.82±24.49	48	32.53±20.85	<0.001	8	60.05±30.4	67	16.45±15.58	0.005
**Age** (years)											
	≤ 44	1,446	70.95±24.76	248	28.87±17.98	<0.001	118	66.98±25.44	168	16.35±11.15	<0.001
	45~54	1,902	68.26±24.48	390	29.46±18.18	<0.001	266	60.66±24.07	362	18.09±15.33	<0.001
	55~64	1,164	64.60±24.45	271	32.47±22.26	<0.001	229	58.31±24.60	242	19.22±16.72	<0.001
	65~74	861	68.41±25.35	315	32.39±23.43	<0.001	142	59.84±25.03	215	20.37±16.96	<0.001
	≥ 75	456	64.53±24.63	427	29.52±22.19	<0.001	89	55.32±22.78	382	16.59±16.58	<0.001
**Mean age** (y±sd)		53.85±12.92		61.84±15.71		<0.001	57.14±12.38		62.24±15.65		<0.001
**Monthly salary** (NT dollars)											
	Low-income	71	65.50±23.65	15	26.80±21.27	<0.001	13	67.75±30.06	24	14.05±9.57	<0.001
	≤ 17280	372	70.56±26.51	83	29.42±21.25	<0.001	51	59.31±27.09	66	15.25±13.58	<0.001
	17281~22800	2,961	71.73±25.18	966	31.21±22.08	<0.001	430	62.99±26.35	708	19.09±16.66	<0.001
	22801~28800	1,077	60.03±20.33	282	26.83±16.36	<0.001	182	55.68±18.48	285	16.53±14.00	<0.001
	28801~36300	479	63.25±24.81	106	34.45±23.42	<0.001	69	54.44±21.28	101	18.37±16.69	<0.001
	36301~45800	432	65.06±25.10	97	31.53±19.30	<0.001	49	59.80±26.69	82	18.83±16.66	<0.001
	≥ 45801	437	67.75±24.75	102	29.35±19.63	<0.001	50	60.05±23.62	103	16.44±15.06	<0.001
**Urbanization level**											
	Level 1	1,716	67.07±24.75	442	30.98±21.08	<0.001	226	58.06±23.49	352	17.69±16.03	<0.001
	Level 2	1,820	67.82±25.15	485	29.76±20.84	<0.001	281	59.75±24.59	415	17.56±15.05	<0.001
	Level 3	932	70.09±24.61	266	30.50±20.40	<0.001	150	66.25±26.30	255	18.12±16.33	<0.001
	Level 4	810	68.22±24.47	233	31.83±21.68	<0.001	110	59.31±23.14	200	18.17±16.23	<0.001
	Level 5	130	65.73±23.81	49	28.51±19.01	<0.001	13	55.95±19.33	27	19.29±15.29	<0.001
	Level 6	197	67.16±24.35	91	29.41±22.37	<0.001	25	56.28±28.04	65	18.83±15.03	<0.001
	Level 7	224	67.21±24.66	85	29.72±21.52	<0.001	39	59.16±25.26	55	20.92±17.55	<0.001
**CCI score**											
	≤ 3	5,492	68.05±24.72	1,352	31.32±21.12	<0.001	743	59.73±24.34	989	19.18±16.20	<0.001
	4~6	282	65.86±26.20	223	28.03±19.95	<0.001	70	61.25±26.64	199	17.06±15.61	<0.001
	≥ 7	55	66.54±23.61	76	21.88±19.74	<0.001	31	69.34±24.83	181	12.70±12.43	<0.001
**Other Catastrophic Illness**											
	No	5,692	67.97±24.76	1,558	30.75±21.06	<0.001	826	60.34±24.61	1,304	18.25±15.98	<0.001
	Yes	137	66.01±25.75	93	25.19±19.47	<0.001	18	54.10±23.53	65	13.24±11.02	<0.001

N = number, m = mean, sd = standard deviation, y = years, NT dollars = New Taiwan dollars.

The survival hazard ratios based on cancer stages (Table 4) showed that the patients who started treatment >180 days were 1.44 (95% CI: 1.07–1.94, P < 0.05) and 1.45 (95% CI: 1.12–1.88, P < 0.05) times as likely to die in stages I and II and stages III and IV, respectively, compared with those who started their treatment within 90 days. Compared with the reference group, in terms of patient health status, high CCI scores and other catastrophic illnesses significantly affected the risk of death, regardless of cancer stage. In terms of hospital factors, the patients treated at hospitals with low service volumes were more likely to die regardless of cancer stage (P < 0.05).

**Table 4:** Cox proportional hazard model: Factors associated with survival.

Variables	Stage I&II			Stage III&IV		
	HR	95% CI	P value	HR	95% CI	P value
**Interval from cancer diagnosis to treatment**						
≤ 90 days (ref.)						
91~180 days	1.31	0.94–1.82	0.107	1.03	0.66–1.59	0.907
>180 days	1.44	1.07–1.94	0.015	1.45	1.12–1.88	0.005
**Age** (years)						
≤ 44 (ref.)						
45~54	1.16	0.99–1.37	0.064	0.94	0.78–1.13	0.485
55~64	1.30	1.09–1.55	0.003	0.77	0.63–0.95	0.012
65~74	1.78	1.50–2.10	< .0001	0.84	0.68–1.03	0.100
≥ 75	3.51	2.98–4.13	< .0001	1.62	1.35–1.95	< .0001
**Monthly salary** (NT dollars)						
Low-income (ref.)						
≤ 17280	1.17	0.67–2.03	0.587	0.92	0.57–1.48	0.719
17281~22800	1.26	0.75–2.10	0.383	0.88	0.58–1.32	0.527
22801~28800	1.25	0.74–2.11	0.403	0.94	0.61–1.43	0.762
28801~36300	1.09	0.63–1.88	0.753	0.94	0.60–1.48	0.797
36301~45800	1.00	0.58–1.73	0.995	0.91	0.57–1.44	0.676
≥ 45801	0.95	0.55–1.64	0.856	1.03	0.65–1.62	0.908
**Urbanization**						
Level 1 (ref.)						
Level 2	1.01	0.88–1.15	0.916	1.00	0.87–1.15	0.990
Level 3	1.02	0.88–1.19	0.759	1.05	0.89–1.24	0.551
Level 4	0.91	0.78–1.08	0.279	1.05	0.88–1.26	0.573
Level 5	1.00	0.74–1.35	0.977	1.24	0.83–1.85	0.286
Level 6	1.22	0.97–1.54	0.095	1.16	0.88–1.53	0.278
Level 7	1.07	0.85–1.36	0.560	0.86	0.64–1.15	0.310
**CCI score**						
≤ 3 (ref.)						
4~6	1.92	1.66–2.23	< .0001	1.40	1.20–1.64	< .0001
≥ 7	3.23	2.55–4.08	< .0001	2.27	1.92–2.68	< .0001
**Other Catastrophic Illness**						
No (ref.)						
Yes	1.63	1.32–2.02	< .0001	1.50	1.17–1.94	0.002
**Hospital level**						
Medical centers (ref.)						
Regional hospitals	0.95	0.80–1.13	0.570	1.02	0.86–1.21	0.848
District hospitals	1.25	0.76–2.04	0.380	1.53	0.97–2.43	0.068
Others	0.99	0.44–2.27	0.985	1.32	0.54–3.25	0.540
**Hospital ownership**						
Public (ref.)						
Private	0.92	0.82–1.04	0.173	0.76	0.67–0.87	< .0001
**Hospital services volume**						
Low (ref.)						
Middle	0.79	0.67–0.93	0.005	0.78	0.66–0.93	0.006
High	0.64	0.53–0.77	< .0001	0.72	0.60–0.88	0.001

HR = hazard ratio, CI = confidence interval, ref. = reference, NT dollars = New Taiwan dollars.

**Fig 1:**
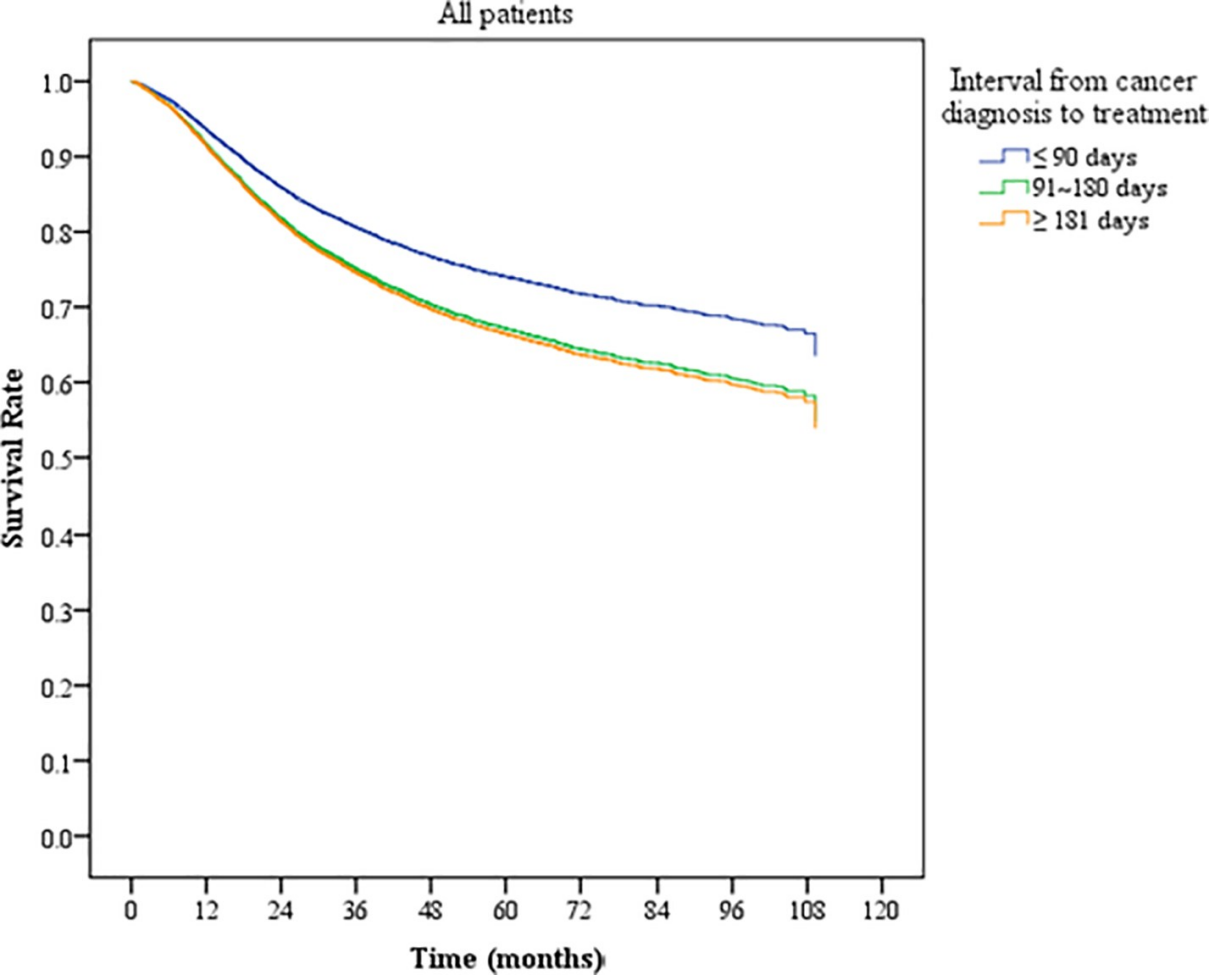
Kaplan–Meier curves of cervical cancer patients with different cancer stages.

**Fig 2:**
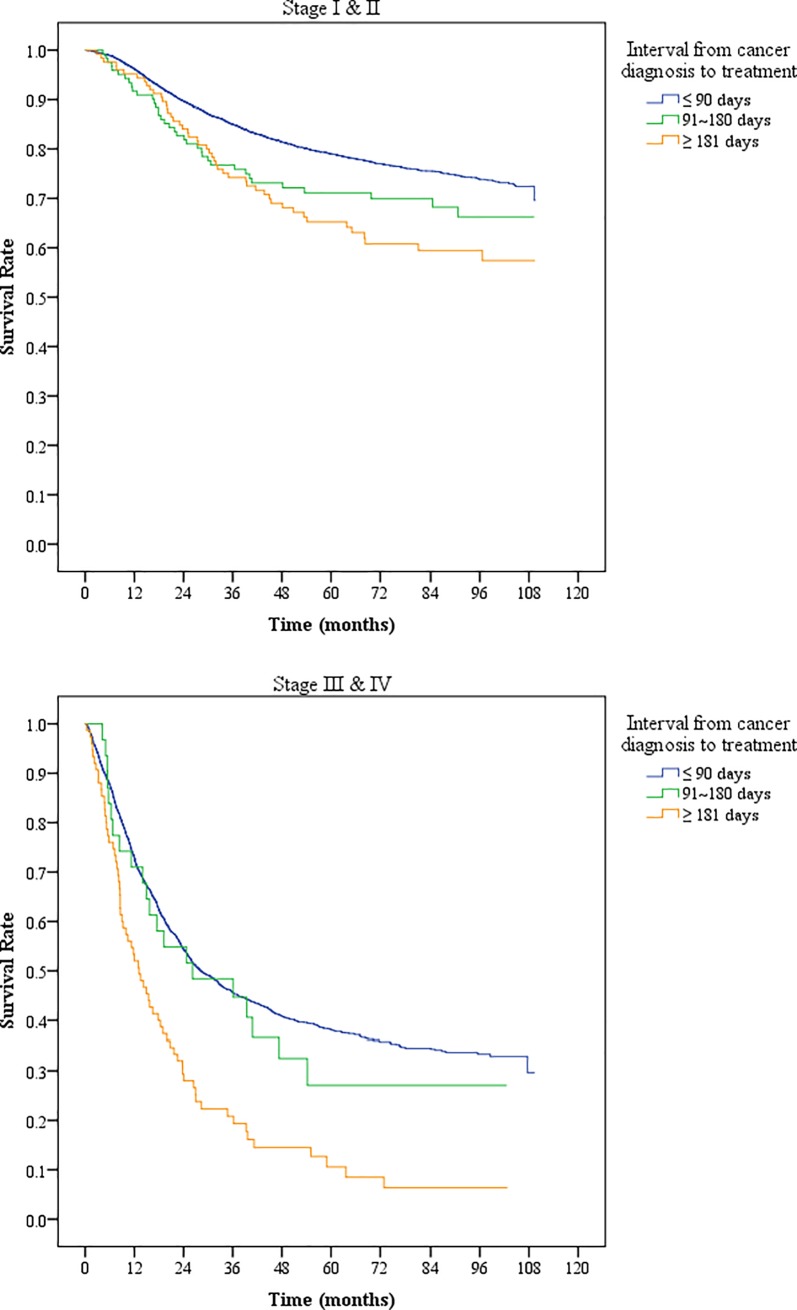
Adjusted survival curves of cervical cancer patients with different cancer stages. Related variables were controlled, including patients’ age, monthly income, urbanization level, CCI score, other catastrophic illnesses, hospital level, hospital ownership, and hospital service volume.

In Kaplan–Meier analysis, the patients who started treatment after a 90-day interval from diagnosis had a lower overall survival rate than did those who started treatment within 90 days (Fig 1). As shown by Kaplan–Meier curves, the difference between the survival rates of the two groups was significant (*P* < 0.05) and increased with time. When the patients were stratified according to their initial tumor stage, namely stages I and II and stages III and IV, the time interval from diagnosis to treatment remained a significant prognosticator in those who started treatment >180 days after controlling for related variables (Fig 2).

## Discussion

The results of this study showed that patients generally sought treatment within 90 days after diagnosis. Our analysis revealed that cervical cancer patients with advanced age (≥65 years) were more likely to have a longer interval between cancer diagnosis and treatment (Table 1). Patients, especially elderly patients, diagnosed with cancer often do not realize the seriousness of their conditions before consultation, and the fear of cancer usually influences their help-seeking behavior after initial consultation. [22] Studies have reported comparable observations and have stated that the rate or treatment refusal increased with an increase in the age of cervical cancer patients. [23–27] Su et al. [3] indicated that the decline in cervical cancer mortality after screening program initiation in Taiwan since 1995 were not observed in elderly women because of treatment delays during the initial implementation of the screening program. Shen et al. [28] also reported that cervical cancer patients aged >65 years at diagnosis were more likely to delay treatment. Unlike Shen’s study, which arbitrarily used 4 months as treatment delay, we defined “cutoff points for first-time treatment” by using a monthly schedule to calculate the survival hazard ratio and divided the patients into three groups.

Some studies have indicated that economic factors may affect the prognosis status. Gong et al. [29] found that patients who lived in an urban area had more favorable prognosis than those who lived in a rural area. Eggleston et al. [30] and Schrijvers et al. [31] have concluded that compared with women with low socioeconomic status (SES), those with high SES exhibited higher survival rates. In our study, after controlling for other variables in our regression model, we found that the urbanization level of the residential area and monthly income did not affect survival rates in the patients under treatment (*P* > 0.05, Table 2). The following explanation can be provided for the difference between our findings and those of other studies conducted in other countries. Under Taiwan’s NHI program and medical outreach programs for remote areas, the government subsidizes the insurance premiums of economically disadvantaged individuals, thus reducing the barriers to medical care for low-income individuals and minimizing the effects of income on treatment-seeking behavior and treatment delay.

Previous studies have identified multiple factors that affect treatment refusal or discontinuation by cancer patients. These factors include having multiple cancers, [27] an advanced cancer stage, or worsening disease and [26] patients’ state of health, access to pertinent information, attitude toward their disease, interaction with and encouragement from health care staff, and concern about adverse effects of treatment. [7] Using a questionnaire, Tsai et al. [32] examined the reasons of avoiding treatment or interrupted treatment within 4 months among patients with breast, colon, oral, and cervical cancer. The main reasons for treatment delay of cervical cancer patients were fear of surgery, economic burden on the household, concerns about poor life quality after therapy, and lack of companions for treatment. According to the results of our regression analysis (Table 1), cervical cancer patients with higher CCI scores and more advanced cancer stage showed significantly longer intervals between diagnosis and treatment. In addition to cases of cervical cancer with advanced stage, Shen et al. found cases of cervical cancer with unspecified stage, which may have indicated that a proportion of the patients did not wish to undergo further testing for cancer stage determination. Similar cases were also observed in our study and were excluded from the analysis.

Among the types of hospitals surveyed (area hospitals, regional hospitals, and medical centers), treatment may be delayed in smaller hospitals because they lack a comprehensive treatment plan or are unable to efficiently treat large numbers of patients. Other studies have shown that treatment may be delayed in higher level hospitals due to lengthy waiting lists or a lack of physical space for numerous patients. However, our study showed that hospitals with low service volumes negatively affected the interval from diagnosis to treatment. This discrepancy might result from differences in cancer type, which requires intervention by a multidisciplinary team.

Treatment of cervical cancer depends on the stage of the disease. Although cancer stages strongly affect the survival rate, our study revealed that regardless of cancer stage, intervals of >180 days between diagnosis and treatment were associated with a higher likelihood of death than are intervals of <90 days. Some studies [7, 33] have shown that longer waiting times between diagnosis and treatment and from the initial visits to surgical intervention in cervical cancer patients were not associated with worse survival. Perri et al. [7] analyzed 321 patients newly diagnosed with cervical cancer between 1999 and 2010. The time from diagnosis to treatment was categorized in three groups that differed in waiting time between diagnosis and treatment initiation: ≤30 days, 30–45 days, and >45 days. The mean age of these 321 subjects was 45 years. With reference to our study results, the conclusion derived from Perri’s study may be confounded by cutoff intervals and the age of subjects.

This study has three strengths. First, this study consisted of cervical cancer patients as participants who were recruited from a nationwide database. Second, this study had a relatively large sample size including 9,693 cervical cancer patients. Third, this study evaluated the effect of time intervals from diagnosis to treatment for cervical cancer patients with different cancer stages. However, this study also has some limitations. The database does not include information on personal lifestyle, education, health behavior, medical knowledge, and family support. The study could not estimate disease-specific survival because no information on the cause of death is available in the database.

## Conclusions

Our study revealed that cervical cancer patients who received treatment between 90 and 180 days or >180 days had a significantly higher death risk than did those who received treatment <90 days. Some of our results are inconsistent with those of studies conducted in other countries; for example, economic status and urbanization level do not affect survival because the government subsidizes the insurance premiums of economically disadvantaged individuals under Taiwan’s NHI program and medical outreach programs for remote areas.

Our study also showed that patients aged >65 years tended to have their treatment at an interval of >90 days, which increased the risk of death compared with other age groups. The attitudes, beliefs, and social contexts of cancer patients affect their treatment-seeking behaviors. Elderly patients might request additional counseling due to the presence of comorbidities or their attitudes toward cancer, which delay early treatment. Therefore, the government should advocate the merits of the referral system for cancer treatment or improve quality assurance for cancer diagnoses across different types of hospitals. Health authorities should also educate patients or use a case manager to encourage prompt treatment within 90 days, particularly in high-risk groups, such as patients aged >65 years, those with high CCI scores, and those with preexisting catastrophic illnesses or injuries, to reduce mortality risk.
